# Low sensitivity of the fourth‐generation antigen/antibody HIV rapid diagnostic test Determine™ HIV Early Detect for detection of acute HIV infection at the point of care in rural Eswatini: a diagnostic accuracy study

**DOI:** 10.1002/jia2.26517

**Published:** 2025-06-28

**Authors:** Iza Ciglenecki, Nombuso Ntshalintshali, Esther Mukooza, Skinner Lekelem, Mpumelelo Mavimbela, Sindisiwe Dlamini, Lenhle Dube, Nomvuyo Mabuza, Melat Haile, Tom Ellman, Antonio Flores, Olivia Keiser, Sindy Matse, Roberto de la Tour, Alexandra Calmy, Bernhard Kerschberger

**Affiliations:** ^1^ Médecins Sans Frontières (MSF) Geneva Switzerland; ^2^ Institute of Global Health University of Geneva Geneva Switzerland; ^3^ Médecins Sans Frontières (MSF) Mbabane Eswatini; ^4^ Eswatini National AIDS Programme (ENAP), Ministry of Health Mbabane Eswatini; ^5^ National Reference Laboratory (NRL) Ministry of Health Mbabane Eswatini; ^6^ Southern Africa Medical Unit (SAMU) Médecins Sans Frontières Cape Town South Africa; ^7^ University Hospital of Geneva Geneva Switzerland

**Keywords:** acute HIV infection, HIV infection, diagnostic testing, rapid diagnostic test, sub‐Saharan Africa, HIV

## Abstract

**Introduction:**

The diagnosis of acute HIV infection (AHI) is challenging in routine settings because it cannot be detected by routine third‐generation antibody rapid diagnostic tests (RDTs). The current fourth‐generation antibody/antigen RDT, Determine™ HIV Early Detect, has demonstrated high sensitivity in laboratory studies, but field evaluations at the point of care are lacking. We nested a diagnostic accuracy study within a larger study of the burden of sexually transmitted infections in rural Eswatini.

**Methods:**

Adults were enrolled at six routine HIV testing sites (HTS) in the Shiselweni region between June 2022 and April 2023. Determine™ HIV Early Detect was performed by HTS counsellors in parallel with routine HIV testing using a finger‐prick blood sample. The reference test was HIV viral load (VL) in the plasma sample, performed on the Xpert platform in the central laboratory. AHI was defined as a negative or discordant HIV test result according to the national serial RDT algorithm and an HIV VL >10,000 copies/ml, or two consecutive HIV VL measurements between the lower limit of detection (40 copies/ml) and 10,000 copies/ml. Established HIV infection was defined as a positive serial RDT test, and overall HIV infection as either established HIV infection or AHI.

**Results:**

One thousand one hundred and sixty‐three participants had all test results available; 49 (4.2%) were diagnosed with HIV (39 with established HIV according to the serial RDT algorithm and 10 with AHI). AHI prevalence among participants with HIV negative or discordant routine RDT results was 0.9% (10/1124). The sensitivity of Determine™ HIV Early Detect to detect overall HIV infection was 83.7% (95% CI 70.3–92.7) and to detect AHI was 20% (95% CI 2.5–55.6%); the specificity was equally high for both 99.8% (95% CI 99.4–100).

**Conclusions:**

The low sensitivity of Determine™ HIV Early Detect to detect AHI when performed at the point of care using finger‐prick blood samples in our study contrasts with other published evaluations from laboratory settings and highlights the importance of field evaluations of the commonly used diagnostic tests.

## 1 INTRODUCTION

Acute HIV infection (AHI), the brief period between viral acquisition and seroconversion, is marked by high viral load (VL), seeding of viral reservoirs and a high risk of transmission [1, 2, 3]. Diagnosis of AHI before seroconversion is challenging, as it is not detected by routine third‐generation rapid diagnostic tests (RDTs) and the prevalence of AHI is low in routine settings. High‐income settings use fourth‐generation immune‐assays, but those are rarely available in resource‐limited settings. Point‐of‐care molecular platforms that can detect HIV VL such as Xpert are widely available but remain expensive.

Fourth‐generation antigen/antibody RDTs may offer a practical alternative. Determine™ HIV Early Detect (Abbott Diagnostic Medical Co. Ltd), previously Alere HIV Combo and Determine HIV ULTRA, is an immunochromatographic test for the qualitative detection of free HIV‐1 p24 antigen in addition to the antibodies to HIV‐1 and HIV‐2 [4]. The test was prequalified by WHO in 2016. Laboratory‐based studies reported sensitivity from 28% to 100% [5, 6, 7, 8, 9, 10, 11, 12, 13, 14]. However, there is limited data on the diagnostic performance of the test when performed on finger‐prick blood in routine settings.

Here, we report the results of a field performance evaluation of Determine™ HIV Early Detect, nested within a larger cross‐sectional study of the burden of sexually transmitted infections in Eswatini.

## 2 METHODS

### 2.1 Study design and setting

The study took place in the predominantly rural Shiselweni region, with high HIV prevalence among adults ≥15 years (24.8% in 2021) [15] and achieved global 95‐95‐95 targets on HIV testing, treatment and viral suppression [16]. The study was conducted at four public health facilities (one secondary‐level facility and three primary clinics) and two community HIV care sites.

Adults aged ≥18 years attending HIV testing sites (HTS) at the study sites between June 2022 and April 2023 were eligible for the diagnostic accuracy sub‐study. Consenting participants completed a questionnaire, underwent HTS, had blood and urine samples taken for further diagnostics.

### 2.2 HIV diagnostics

Routine HIV testing was performed by HTS counsellors on finger‐prick blood using the national serial RDT algorithm (Determine™ and Uni‐Gold™). The index test Determine™ HIV Early Detect was performed in parallel to routine HIV testing on finger‐prick blood, using blood from the same or second finger‐prick if necessary. The test was read after 20 minutes by the same HTS counsellor. HTS counsellors received specific training to perform and interpret the test and were supervised by a trained laboratory technician.

Venous blood was collected at the same time in tubes with ethylenediaminetetraacetic acid (EDTA) and sent to the central laboratory or spined on site (if same‐day transfer not possible). Diagnostic HIV VL testing was performed at the Nhlangano laboratory on plasma, using the Xpert platform (Xpert® HIV‐1 Viral Load).

Participants were informed about their HIV status based on the results of the routine serial HIV RDT and were offered immediate anti‐retroviral treatment (ART) initiation and partner notification. Participants diagnosed with AHI based on HIV VL were informed once the results were available, usually within a week, and invited to link to care. Participants who tested negative were offered HIV prevention interventions.

### 2.3 Analysis and statistics

All data were analysed using STATA 18 (StataCorp, TX, USA). Key participant's characteristics were described by HIV status. Categorical variables were summarized as counts and percentages and the distributions of categorical variables between the groups were compared using Fisher's exact test. Comparisons of continuous variables was performed with the Wilcoxon rank‐sum test.

### 2.4 Definitions

Established HIV infection was defined according to the national serial HIV testing algorithm, based on detectable HIV antibodies on first‐line (Determine™) and second‐line (Uni‐Gold™) HIV RDTs. AHI was defined as a negative or discordant result according to the national serial RDT algorithm and an HIV VL >10,000 copies/ml, or two consecutive detectable HIV VL measurements between the lower limit of detection (40 copies/ml) and 10,000 copies/ml to rule out possible false positive VL results and align with a previous study in the same setting [14, 17]. Serial HIV RDT algorithm negative or discordant samples with undetectable HIV VL were considered negative. Overall HIV infection was defined as either established HIV infection or AHI.

Determine™ HIV Early Detect was considered positive if reactive on the antibody line alone, the antigen line alone or both lines simultaneously.

### 2.5 Determine™ HIV Early Detect performance

The performance of Determine™ HIV Early Detect to detect overall HIV infection was calculated in participants with paired routine RDT, VL and index test results available and the performance to detect AHI in participants who tested negative or discordant on the standard serial algorithm and had VL and index test results available. We calculated diagnostic accuracy indicators and positive (PPV) and negative predictive values (NPV) with 95% confidence intervals (CI) for Determine™ HIV Early Detect to detect overall HIV versus the serial RDT algorithm or positive HIV VL as the reference, and for AHI with HIV VL as reference.

### 2.6 Sample size

The sample size for diagnostic accuracy was estimated at 1201 participants to allow detection of 70% sensitivity with a 15% precision at 95% confidence level, assuming 3% AHI prevalence, based on the prevalence in the same setting [18]. The sensitivity assumption was based on the manufacturer's information and previous studies [5, 6, 12, 19].

### 2.7 Ethical considerations

All participants provided written informed consent prior to enrolment. The study was approved by the Eswatini Health and Human Research Review Board (EHHRRB096/2021) and the MSF Ethics Review Board (ID:2154).

## 3 RESULTS

Of 1195 participants with negative or unknown HIV status who underwent HTS, 22 were excluded from the analysis due to missing final HIV status ascertainment (four missing Determine™ and 18 VL results). Index test results were missing for a further 10 participants. Finally, data were retained for the diagnostic accuracy analysis for 1163 (97.3%) participants with a median age of 27 years (IQR 22–33), of whom 709 (61%) were women (Table 1).

**Table 1:** Key characteristics of patients enrolled in the study, by final HIV status

	Total	HIV positive	Established HIV	AHI	HIV negative	
	*N* = 1124	*N* = 49	*N* = 39	*N* = 10	*N* = 1114	*p*‐value*
**Enrolment site**						
Nhlangano health centre	354 (30.4%)	15 (30.6%)	12 (30.1%)	3 (30%)	339 (30.4%)	0.286
Factory clinic	166 (14.3%)	10 (20.4%)	7 (18.0%)	3 (30%)	156 (14.0%)	
Zombodze primary clinic	135 (11.6%)	9 (18.4%)	8 (20.5%)	1 (10%)	126 (11.3%)	
Gege primary clinic	107 (9.2%)	6 (12.2%)	4 (10.3%)	2 (20%)	101 (9.1%)	
Nhlangano fixed site	211 (18.1%)	5 (10.2%)	5 (12.8%)	0 (0%)	206 (18.5%)	
Lavumisa fixed site	190 (16.3%)	4 (8.2%)	3 (7.7%)	1 (10%)	186 (16.7%)	
**Sex**						
Female	709 (61.0%)	38 (77.5%)	29 (74.4%)	9 (90%)	671 (60.2%)	0.037
Male	454 (39.0%)	11 (22.5%)	10 (25.6%)	1 (10%)	443 (39.8%)	
**Age**						
Age, median (IQR)	27 (22–33)	28 (25–35)	28 (25–35)	22.5 (19–27)	27 (22–33)	
Age: 18–29 years	727 (62.5%)	30 (61.2%)	21 (53.8%)	9 (90%)	697 (62.6%)	0.109
Age: 30 years or more	436 (37.5%)	19 (38.8%)	18 (46.2%)	1 (10%)	417 (37.4%)	
**Discordant RDT result**	4 (0.4%)	2 (4.1%)	0 (0%)	2 (20%)	2 (0.2%)	0.001
**HIV VL** (copies/ml), median (IQR)		86,400 (11,700–318,000)	34,200 (3460–146,000)	3.4M (0.75M–10M)		<0.001**

Abbreviations: AHI, acute HIV infection; VL, viral load.

*p‐value comparing established HIV, AHI and HIV negative (Fischer exact) test.

**p‐value comparing established HIV and AHI (Wilcoxon ranksum test).

**Figure 1:**
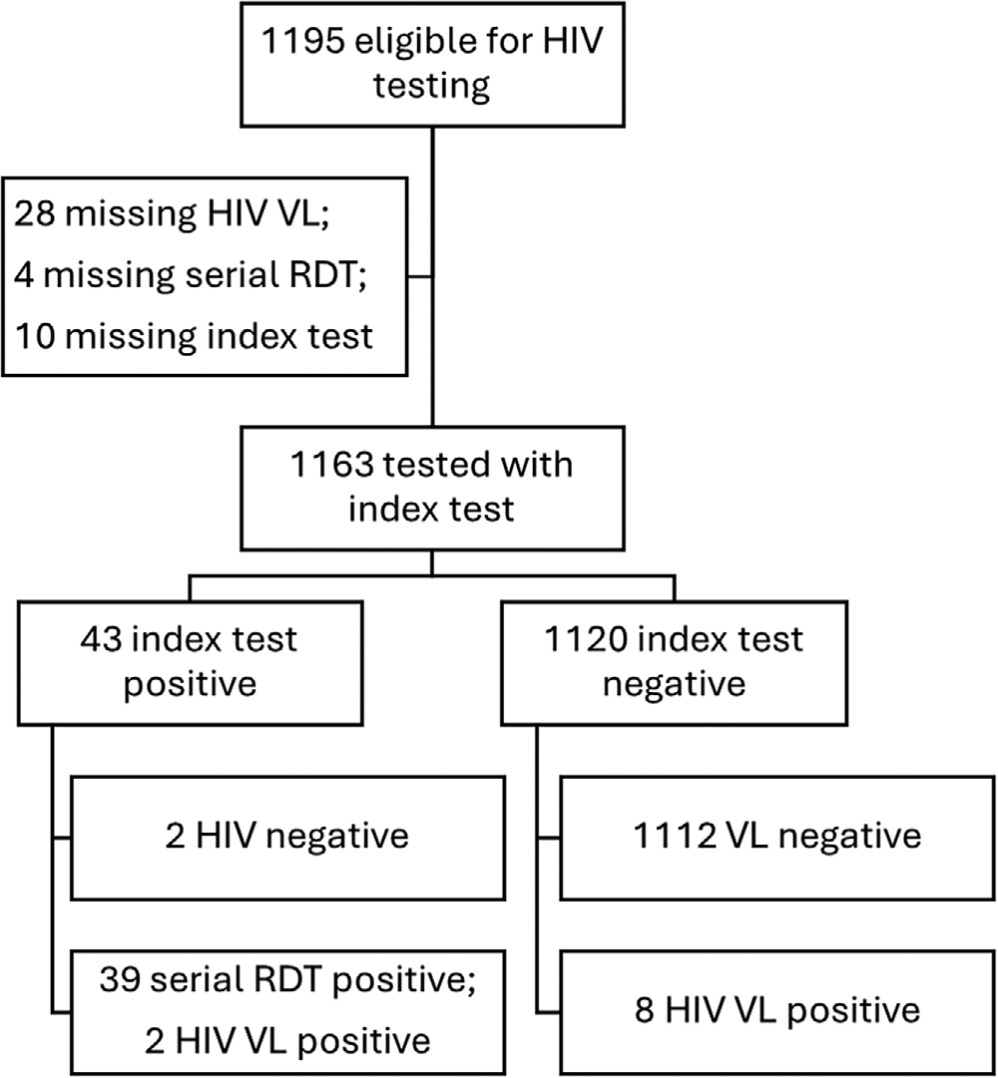
Flowchart for diagnostic accuracy of Determine™ HIV Early Detect to diagnose overall HIV infection and acute HIV infection. Index test, Determine™ HIV Early Detect; RDT, rapid diagnostic test; Serial RDT, Determine™ followed by Unigold™ if Determine™ positive; VL, viral load.

Of 1163 participants, 49 (4.2%) were diagnosed with HIV according to the study definitions: 39 with established HIV and 10 with AHI (Figure 1). Of four discordant RDT results, two had detectable VL and were considered AHI, and two had undetectable VL and were considered HIV negative. Ten (0.9%) among 1124 participants with HIV negative or discordant results on the routine HTS algorithm were diagnosed with AHI.

### 3.1 Diagnostic performance of Determine™ HIV Early Detect

#### 3.1.1 Overall HIV infection

The diagnostic performance of Determine™ HIV Early Detect to detect overall HIV infection and AHI is summarized in Table 2. Determine™ HIV Early Detect correctly detected 41 of 49 overall HIV infections; sensitivity was 83.7% (95% CI 70.3–92.7). Most tests were positive on the antibody line (37/41, 90%), two on the antigen line only and two on both lines. All reactive Determine™ RDT results were also reactive on Determine™ HIV Early Detect. The test was false positive in two cases (specificity 99.8% [95% CI 99.4–100]), one on the antibody line and one on both lines (both non‐reactive Determine™ and undetectable VL). The PPV and NPV were high, 95.3% (95% CI 84.2–99.4) and 99.3% (95% CI 98.6–99.7), respectively.

**Table 2:** Diagnostic accuracy indicators for Determine™ HIV Early Detect (index test) to detect overall HIV infection and acute HIV infection.

	Overall HIV infection		Acute HIV infection	
Index test/VL	Negative	Positive	Negative	Positive
Positive	2	41	2	2
*p24*	*0*	*2*	*0*	*1*
*p24 and AB*	*1*	*2*	*1*	*0*
*AB*	*1*	*37*	*1*	*1*
Non‐reactive	1112	8	1112	8
**Diagnostic performance indicators**				
Sensitivity (95% CI)	83.7% (70.3–92.7)		20% (2.5–55.6%)	
Specificity (95% CI)	99.8% (99.4–100)		99.8% (99.4–100%)	
AUC (95% CI)	0.92 (0.87–0.97)		0.60 (0.47–0.73%)	
PPV (95% CI)	95.3% (84.2–99.4)		50% (6.8–93.2%)	
NPV (95% CI)	99.3% (98.6–99.7)		99.3% (98.6–99.7%)	

*Note*: HIV prevalence 4.3% and acute HIV prevalence 0.9%.

The numbers in italic represent number of infections detected on antigen (p24), antibody (AB) or both lines (p24 and AB).

Abbreviations: AUC, area under the curve; CI, confidence interval; NPV, negative predictive value; PPV, positive predictive value.

#### 3.1.2 Acute HIV infection

Determine™ HIV Early Detect was reactive in four participants with negative (2/1120) or discordant (2/4) routine RDT. Two reactive results (one positive antibody line and one both lines) with negative routine RDT had undetectable VL and were considered false positive and two were confirmed AHI (one positive antigen line and one antibody line), both with discordant routine RDT. The remaining eight AHI were missed by Determine™ HIV Early Detect (sensitivity 20%; 95% CI 2.5–55.6%). The specificity (99.8%, 95% CI 99.4–100%) and NPV (99.3%, 95% CI 98.6–99.7%) were high, but PPV was low (50%; 95% CI 6.8–93.2%).

## 4 DISCUSSION

In this field evaluation of Determine™ HIV Early Detect, we show poor sensitivity (20%) to detect AHI when performed in a routine care setting on finger‐prick blood by HTS counsellors. Although it detected all infections identified by the routine first‐line Determine™, the sensitivity to detect overall HIV infection was lower than expected (83.3%), due to the high proportion of AHI.

The low sensitivity observed contrasts with the results of several laboratory‐based studies of the current version of Determine™ HIV Early Detect, ranging from 28% to 100% [5, 8, 9, 11, 12, 13] and with our earlier study in Eswatini, showing 86% sensitivity in plasma and 79% in venous whole blood [18]. However, another study in Indonesia on finger‐prick samples also showed limited sensitivity: none of the six AHI cases were detected by Determine™ HIV Early Detect (preprint [20]).

Evaluating antigen‐based diagnostic tests for AHI is challenging since the prevalence of AHI in clinical samples is very low, therefore, most studies used stored samples with a known diagnosis [21]. The results of these studies are also difficult to interpret and compare, as they are mostly based on small numbers of AHI cases, with different proportions of AHI stages, and using different reference methods for AHI or seroconversion [9, 21].

We compared the laboratory characteristics of AHI in this study with earlier studies with better sensitivity conducted in the same setting [18]. The 2019 study used the same definitions and diagnostic tools, but the Determine™ HIV Early Detect was performed on plasma or whole blood, in the laboratory by a trained laboratory technician. The AHI prevalence was higher (3.9% for plasma and 4.4% for whole blood samples), and the VL count was approximately 30‐fold lower compared to the current study. This is particularly surprising, as the sensitivity of Determine™ HIV Early Detect to detect antigen correlates with the highest VL [9]. The lower sensitivity in the current study may be explained by participants being detected at an earlier AHI stage, before antigen appearance (Fiebig stage 1), during the “second diagnostic window” due to a drop in p24 antigen levels before antibody detection [22] or during early seroconversion, when antigen/antibody complex formation results in the variable presence of free antigen and/or antibody [23, 24]. Determine™ HIV Early Detect only detects free antigen and/or antibodies [19]; insufficient immune complex disruption was previously described as a major challenge for antigen/antibody RDTs performance [25]. The use of finger‐prick whole blood may further explain the lower sensitivity. Previous studies have shown higher sensitivity of the current version of Determine™ HIV Early Detect when performed in plasma compared to whole blood [18] or in plasma seroconversion panels compared to simulated whole blood [6], and the previous version of the test performed better in serum compared to finger‐prick blood [26]. Field performance may also be influenced by the level of staff training, and environmental conditions, such as temperature, humidity or dust [27]. HIV subtype may play a role, with lower test sensitivity in the contexts where non‐B sub‐types are prevalent [28, 29], as is the case in Eswatini with the predominance of subtype C [30].

Although Determine™ HIV Early Detect detected the same HIV infections as Determine™ (all established HIV infections and two AHI with discordant RDT result), the overall sensitivity to detect HIV infection was only 83.7%, as AHI represented a fifth of all infections, similar to the reduced overall RDTs sensitivity documented in other studies [11, 31]. It is expected that the proportion of AHI among first‐time diagnosed HIV infections will increase, as the frequency of testing increases and more people living with HIV (PLHIV) are aware of their status [31, 32].

Determine™ HIV Early Detect is being used at the point of care in many settings with the hope to increase the detection of AHI [33]. For example, in Barcelona checkpoints, the test detected four additional Ag‐only positive cases with high VL (0.9% of new HIV diagnosis) [34], although the proportion of AHI may be higher in the context of frequent testing [31].

Our study has several limitations. The AHI prevalence was lower than estimated, making diagnostic accuracy results less precise. We used pragmatic definitions of established HIV and AHI, without additional laboratory analysis to differentiate AHI stages. Some infections may have been missed if VL was undetectable and the third‐generation RTD was false negative, that is in the context of retesting among PLHIV receiving ART, or breakthrough infections under pre‐exposure prophylaxis. We did not assess associations between test performance and behavioural or clinical risk factors, as this was beyond the scope of this diagnostic analysis. Despite the training and established standard operating procedures, errors in the conduct of the test were possible. The index test was conducted in parallel to the first‐line RTD, possibly influencing the conduct of either the test or the reading of the results; we did not take photos to confirm the results. Future field studies should include a larger number of AHI cases, investigate the role of sample type and staff qualifications and include qualitative insights to understand the usability of the test at the point of care.

The main strength of the study is its real‐life setting, the conduct of the test on finger‐prick blood by lay HTS counsellors, addressing a major gap in the performance evaluation of this test. The counsellors performing the test were blinded to the results of the reference test, which was done for all tested participants within days of sample collection.

## 5 CONCLUSIONS

The promising and already widely used fourth‐generation antibody/antigen RDT Determine™ HIV Early Detect had poor sensitivity in our study, when performed in a routine HTS setting on finger‐prick blood. Further point‐of‐care field studies are needed to understand its performance in real‐world settings. Our study also highlights the remaining challenge in achieving HIV epidemic control if AHI is not addressed amidst the high levels of HIV status awareness and high ART coverage. Accurate and accessible point‐of‐care RDTs are needed to scale‐up AHI diagnosis in resource‐limited settings.

## COMPETING INTERESTS

MSF provided support in the form of salaries for IC, BK, NN, EM, SL, MH, TE, RT and AF. AC declares unrestricted educational grants from Gilead Sciences, ViiV Healthcare and MSD, paid to the institution. All other authors declare no competing interests.

## AUTHORS’ CONTRIBUTIONS

IC, NN and BK conceived and designed the analysis. BK and EM supervised the study implementation. NN supervised laboratory aspects of the study. BK, EM, SK and NN curated and verified the data. IC and BK performed the analysis and IC wrote the original draft. All authors contributed to the interpretation of the findings and reviewed the paper.

## FUNDING

This study was funded by Médecins Sans Frontières, Operational Center Geneva.

## Data Availability

The minimal dataset underlying the findings of this paper are available on request, in accordance with the legal framework set forth by MSF data sharing policy. MSF is committed to sharing and disseminating health data from its programmes and research in an open, timely, and transparent manner to promote health for populations while respecting ethical and legal obligations towards patients, research participants, and their communities. The MSF data sharing policy ensures that data will be available upon request to interested researchers while addressing all security, legal, and ethical concerns. Readers can contact the MSF generic address data.sharing@msf.org or the corresponding author to request the data that can be shared with researchers subject to the establishment of a data sharing agreement to provide the legal framework for data sharing.
